# Parents' perception of self-advocacy of children with myositis: an anonymous online survey

**DOI:** 10.1186/1546-0096-9-10

**Published:** 2011-06-07

**Authors:** James D Katz, Gulnara Mamyrova, Shilpi Agarwal, Olcay Y Jones, Harriet Bollar, Adam M Huber, Lisa G Rider, Patience H White

**Affiliations:** 1 Division of Rheumatology, The George Washington University, Washington, DC, USA; 2 NIEHS NIH, Bethesda, MD, 20892-1301 USA; 3 Walter Reed Army Medical Center, Washington, DC 20307, USA; 4 IWK Health Centre and Dalhousie University, Halifax, Nova Scotia, B3H 4R2, Canada; 5 Glendale Adventist Family Medicine Residency, Glendale, CA 91205, USA; 6 Cure JM, Wilmette, IL 60091, USA

## Abstract

**Background:**

Children with complex medical issues experience barriers to the transition of care from pediatric to adult providers. We sought to identify these barriers by elucidating the experiences of patients with idiopathic inflammatory muscle disorders.

**Methods:**

We collected anonymous survey data using an online website. Patients and their families were solicited from the US and Canada through established clinics for children with idiopathic inflammatory muscle diseases as well as with the aid of a nonprofit organization for the benefit of such individuals. The parents of 45 older children/young adults suffering from idiopathic inflammatory muscle diseases were surveyed. As a basis of comparison, we similarly collected data from the parents of 207 younger children with inflammatory muscle diseases. The survey assessed transition of care issues confronting families of children and young adults with chronic juvenile myositis.

**Results:**

Regardless of age of the patient, respondents were unlikely to have a designated health care provider assigned to aid in transition of care and were unlikely to be aware of a posted policy concerning transition of care at their pediatrician's office. Additionally, regardless of age, patients and their families were unlikely to have a written plan for moving to adult care.

**Conclusions:**

We identified deficiencies in the health care experiences of families as pertain to knowledge, self-advocacy, policy, and vocational readiness. Moreover, as children with complex medical issues grow up, parents attribute less self-advocacy to their children's level of independence.

## Background

Children with complex medical needs endure noteworthy barriers to the process of transitioning from adolescent to adult healthcare [1]. Knowledge deficits about medical history and medicine regimens are just two examples of such barriers that must be addressed [2]. Other concerns of families with teenagers who are close to adult age may include insurability, employability, and independent living [3]. By the same token, physicians may have trepidation implementing transition of care owing to poor academic preparation in this regard. For example, current ACGME requirements for pediatric and internal medicine training do not include curricula in transitioning care [4]. Therefore, it is not surprising that adolescents anticipating transfer to adult care providers have anxiety about this process and need guidance about such issues as the timing of transfer of care to an adult health care provider [5]. It has been recommended that subspecialty pediatric providers should anticipate such concerns and implement programs to facilitate transitioning of care [5]. The primary objective of our study is to characterize the transition experience of families of children with chronic idiopathic inflammatory myopathies (IIM) and to identify areas for improvement.

## Methods

The George Washington University Myositis Center treats both children and adults with IIM. An on-line survey was developed aimed at anonymously assessing transition of care issues confronting families of children and young adults with chronic juvenile myositis. Local IRB approval was secured from The George Washington University Office of Human Research prior to the implementation of the project. In addition to soliciting the involvement of our patient population and that of the Environmental Autoimmunity Group, NIEHS, we were able to broaden our reach both through international collaboration (Canada) and through partnering with Cure JM, a non-profit organization dedicated to the cure of juvenile myositis http://www.curejm.com/. Survey responses were collected on-line and anonymously between 4/1/09 and 12/30/10. We collected survey responses from 252 parents, of whom 45 had children in the target transition age range (15.1 years to 21 years old). Subspecialty pediatrician input concerning an individual's diagnosis (for a given respondent) was not a feature of this study design as it would force violation of anonymity. In keeping with anonymity practices, geographical stratification of responses was not possible in our analysis. However, given that the major recruiting centers (located in the Greater Washington DC area) are international referral sites, it is safe to assume that survey respondents do not reflect US mid-Atlantic or East Coast transitioning practices alone. Further more, since these clinics are nonprofit and free to families, it cannot be said that respondents are limited to higher socioeconomic classes. Survey responses were collected through a commercial online portal http://www.surveymonkey.com/. Uploading survey data to a standard spreadsheet enabled statistical analysis. Statistical analyses were performed on the parents' responses as reported here by using GraphPad InStat version 3.00 for Windows 95, GraphPad Software, San Diego California USA, http://www.graphpad.com. Fischer's exact test was used to calculate P values for 2 × 2 tables. A p-value less than or equal to 0.05 was considered significant.

## Results

Forty-five surveys were analyzed representing teens and young adults between the ages of 15.1 and 21 (Table 1). The choice of age 15 as the cut off of interest for transition of care is based upon the results of a survey of primary care pediatricians published elsewhere[13]. Seventy-eight percent of respondents were female patients. The majority of young adults surveyed (87%) carry a diagnosis of juvenile dermatomyositis and satisfy probable to definite Peter and Bohan criteria for confirming a diagnosis of myositis. In order to validate the diagnosis of juvenile myositis, we specifically collected data on the disease criteria that these individuals satisfied. The majority of respondents confirmed the presence of one or more of the following (data not shown): weakness (91%), rash (Gottron's 76%; heliotrope 64%), or abnormal muscle enzymes (82%). Fifty-eight percent reported a history of an abnormal muscle MRI. Moreover, at the time of the survey 29% were presently unable to complete an independent sit up, 20% were unable to climb stairs without assistance, and 12% could not stand from a seated position without help (Table 2). Four children were reported to have swallowing problems.

**Table 1:** Demographic characteristics of pediatric myositis patients

Characteristics	Patients N (%)*	
	
	≤ 15.0 years of age	> 15.0 years of age
Currently living in		
United States	178 (86.0)	41(91.1)
Canada	29 (14.0)	4 (8.9)
Age (y.o.)		
< 3.0	10 (4.8)	
3.1-6.0	47 (22.7)	
6.1-9.0	57 (27.5)	
9.1-12.0	55 (26.6)	
12.1-15.0	38 (18.4)	
15.1-18.0		20 (44.4)
18.1-21.0		25 (55.6)
School grade		
Pre-K	27 (13.5)	
Kindergarten	18 (9.0)	
1^st ^grade	25 (12.5)	
2^nd ^grade	16 (8.0)	
3^rd ^grade	22 (11.0)	
4^th ^grade	13 (6.5)	
5^th ^grade	24 (12.0)	
6^th ^grade	13 (6.5)	
7^th ^grade	12 (6.0)	
8^th ^grade	21 (10.5)	
9^th ^grade	8 (4.0)	1 (2.2)
10^th ^grade	0 (0.0)	5 (11.1)
11^th ^grade	1 (0.5)	8 (17.8)
12^th ^grade		6 (13.3)
Beyond high school		25 (55.6)
Gender		
Female	141 (68.1)	35 (77.8)
Male	66 (31.9)	10 (22.2)
Age at the time of diagnosis of juvenile myositis (y.o.)		
< 3.0	39 (18.9)	0 (0.0)
3.1 - 6.0	89 (43.2)	2 (4.4)
6.1 - 9.0	40 (19.4)	6 (13.3)
9.1 - 12.0	31 (15.0)	10 (22.2)
12.1 - 15.0	7 (3.4)	8 (17.8)
15.1 - 18.0.	0 (0.0)	15 (33.3)
18.1 - 21.0	0 (0.0)	4 (8.9)
Diagnosis		
Juvenile dermatomyositis (JDM)	202 (97.6)	39 (86.7)
Juvenile polymyositis (JPM)	3 (1.4)	4 (8.9)
Other forms of juvenile myositis	2 (1.0)	2 (4.4)

*N = 207 for patients ≤ 15.0 years of age; N = 45 for patients > 15.1 years of age.

**Table 2:** Myositis disease severity characteristics at the time of the survey*

Survey item	Patients N (%)**	
	
	≤ 15.0 years of age	> 15.0 years of age
Right now, can your child do a sit-up without help?	142 (68.6)	32 (71.1)
Right now, can your child climb the stairs without help or using support?	168 (81.2)	36 (80.0)
Right now, can your child stand from a seated position without assistance or support?	179 (86.5)	35 (77.8)
Right now, does your child have a swallowing problem?	12 (5.8)	4 (8.9)

Patients answering in the affirmative

*The reminder of the parents answered "Do not know".

**N = 207 for patients ≤ 15.0 years of age; N = 45 for patients > 15.1 years of age.

As expected, older children (> 15.1 years of age) were likely to have a job outside the home, to be responsible for weekly household chores, to know how to contact the doctor and to know how to refill medications (Table 3). Older teens knew the names of their medications and were deemed capable of explaining their disease to family or friends. Sixty-seven percent knew what health changes required medical attention. Only slightly more than half (51%) were deemed responsible enough to take medications without being reminded and only 20% actually scheduled their own physician appointments. Older children were likely to know the name of their health insurance coverage plan (69%) (Table 3). However, less than 40% reported receiving help with transition-related knowledge or tasks and, in the case of creating a written plan for moving to adult care, only 7% had such a draft (Table 3).

**Table 3:** Level of knowledge of the disease, transition-related knowledge, and experience of families, stratified by age of pediatric myositis patients

Survey Item	Patients N (%)*		P
		
	≤ 15.0 years of age	> 15.0 years of age	
Does your child have a paying or volunteer job?	5 (2.4)	25 (55.6)	< 0.0001

Is your child responsible for any household chores?	109 (52.7)	37 (82.2)	0.0002

Does your child know her/his doctor's name?	172 (83.1)	45 (100.0)	0.0013

Does your child know where to get her/is doctor's phone number?	59 (28.5)	35 (77.8)	< 0.0001

Does your child make her/his own appointments?	0 (0.0)	9 (20.0)	< 0.0001

Does your child take her/his medications without being reminded?	26 (12.6)	23 (51.1)	< 0.0001

Does your child know the names of the medicine she/he takes?	89 (43.0)	35 (77.8)	< 0.0001

Does your child know what to do to take care of her/himself?	37 (17.9)	34 (75.6)	< 0.0001

Does your child know what changes in her/his health require medical attention?	69 (33.3)	30 (66.7)	< 0.001

Can your child explain her/his disease to her/family or friends?	78 (37.7)	38 (84.4)	< 0.0001

Does your child know how to refill her/his own medications/	15 (7.2)	28 (62.2)	< 0.0001

Does your child see the physician alone?	4 (2.0)	9 (20.0)	< 0.0001

At what age do you believe your child could see the physician alone?			
9.1-12.0	9 (4.4)	0 (0.0)	0.3694
12.1-15.0	30 (14.6)	0 (0.0)	0.0038
15.1-18.0	84 (40.8)	11 (27.3)	0.0431
18.1-21.0	70 (34.0)	18 (39.4)	0.4915
> 21.1	13 (6.3)	16 (33.3)	< 0.0001

Has your doctor talked with your child to see a doctor who treats adults?	5 (2.4)	17 (37.80	< 0.0001

Does your child know the name of health insurance she/he currently has?	31 (15.2)	31 (68.9)	< 0.0001

Is there a health care provider who helped you and your child with transition to adult health care provider?	14 (6.9)	10 (22.2)	0.0039

Do you have a portable up-to-date copy of your child's medical records?	76 (37.4)	21 (46.7)	0.3111

Is there a posted policy to see in your pediatric doctor's office states when you are expected to transition?	12 (5.9)	4 (8.9)	0.5

Do you have written plan to moving to adult healthcare?	3 (1.4)	3 (6.7)	0.0720

*N = 207 for patients ≤ 15.0 years of age; N = 45 for patients > 15.1 years of age.

**Figure 1:**
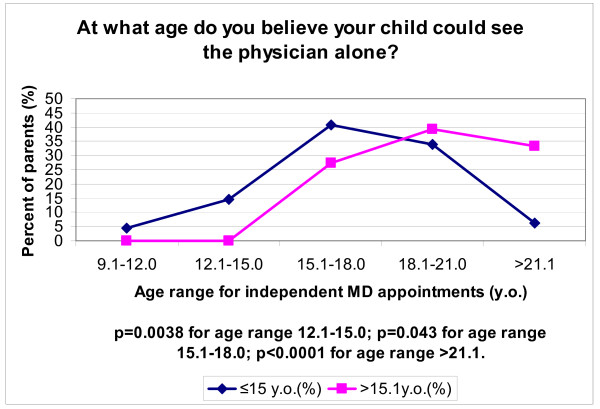
**Parents' perception stratified on age**.

For any given age > 15, 27% or more of parents felt that older children could see the physician alone. However, only 20% of such patients actually did see the doctor alone (Table 3). When asked to identify an age at which older children could see the physician alone, 27% of parents chose the age range of 15.1 - 18 years, while 39% chose the age range of 18.1 - 21 years. Comparing this to the parents of younger children, there was a clear shift in perception of the appropriate age range for independent doctor visits (shifted to an earlier age). In other words, the older the child, the older the parents tended to set the age at which they believed that children should see the doctor alone. For example, parents of younger children were more likely to choose a lower age range of 12.1 - 15 years in answer to this question (p = 0.038) (Figure 1). Significant findings in this manner were similarly obtained for age ranges 15.1-18 and for ages greater than 21.

With regard to transitioning to adult provider-based care, we found that regardless of age of the patient, respondents were unlikely to have a designated health care provider assigned to aid in transition of care and were unlikely to be aware of a posted policy concerning transition of care at their pediatrician's office. Moreover, regardless of age, patients and their families were unlikely to have a written plan for moving to adult care (Table 3). Finally, also regardless of age, less than half of respondents were in possession of a current record of their medical file.

## Discussion

One of the aims of transition of care is:

"to promote skills in communication, decision-making, assertiveness, selfcare, and self-advocacy" [6].

To this end, we assessed the knowledge level of adolescents with juvenile myositis. All children know their doctor's names and most (78%) know where to obtain that doctor's phone number (Table 3). Fewer adolescent children (62%) know how to refill medications even though most knew what the names of the medications were (78%). Only 20% made his or her own appointments to see the health care provider.

Some of the key elements for successful transition of care are:

1) Identification of a key person for each individual patient, 2) A transition policy, 3) A flexible policy on timing of events with anticipation of change, 4) A written health care transition plan, 5) The fostering of personal and medical independence and creative problem solving, 6) Liaison personnel in pediatric and adult teams[6].

In this regard, we assessed the transition-related knowledge and support experienced by families. Thirty-eight percent of families reported having discussed transitioning to an adult care provider with their pediatric care provider and 69% of older children could identify the name of their health insurance (Table 3). However, only 22% of families reported access to a transition healthcare professional, 9% of families were aware of a posted policy for transition in the pediatrician's office, and 7% of families had a written plan for moving to adult healthcare. With regard to self-advocacy skills we found that 47% of parents of older patients have a portable and up to date copy of their child's medical records (Table 3); and this was not statistically different when stratified on younger *vs*. older age (Table 3) suggesting that self advocacy does not "mature" with age of the patient. Indeed, it is possible that this finding more likely reflects self-advocacy of the parent rather than the child.

Data collected on 207 patients aged younger than 15.1 years enabled us to augment our understanding of the transition-related knowledge and support as experienced by families in general (Table 3). Here we learned that 37% of families have a portable, up to date medical record for their younger child, 6% have been exposed to a pediatric practice with a posted policy concerning transition of care, and only 1.4% actually have a written plan for moving to adult care again suggesting that support for transitioning in general is lacking in pediatric practices.

Finally, with regard to vocational readiness [7], we found that 56% of adolescents have a paying or volunteer job outside of the home and 82% of adolescents are responsible for household chores on a regular basis (Table 3).

The transition from pediatric to adult care is a vulnerable time for patients with chronic disease [8]. Not only is it a financially insecure time but preliminary data suggests that adherence to prescribed treatment regimens may decrease after this transition [9]. In this regard, it is important to note that there exists significant variability in transition support afforded to young adults with chronic medical conditions [10]. For example, only one third of adolescent hemodialysis centers have in place a transition program to aid youth in the acclimation process to adult care [11]. Similarly, our data demonstrates that low percentages of families of patients with juvenile myositis have had their transition needs proactively addressed. Less than half of our study subjects were in possession of a portable up to date written medical record and few were either aware of a posted policy for transition of care or able to identify a health care worker responsible for aiding in such a transition. In particular, few patients had a written plan for moving to adult care. This is in keeping with the general, but sparse, academic literature in this regard [12]. For example, in a recent survey, 13% of pediatric practices reported having written policies on the transition and transfer of adolescents to adult care [13].

We caution that our data bears the same weaknesses of all anonymous survey research. This includes inability to control for ambiguities inherent in the language used for the survey (such as choosing to use the word 'could' vs. 'should'), as well as deficiencies owing to self-reported data, small sample size, and the inability to verify that the population studied is representative of the greater population of children with myositis. However, in order to validate the self-reported nature of the study subject diagnosis, we concurrently collected data on the clinical manifestations of disease as well as selected aspects of severity of disease. In this way we were able to confirm that the majority of participants had at one point or other manifested generally accepted clinical features of myositis and that some, indeed, represented more severe disease.

Transition readiness may be a source of anxiety for families [14]. Our data confirms this perspective in a unique manner because we find that based upon cross-sectional data, there may be a shift in parents' attitudes as their children mature. Longitudinal data will be needed to confirm that parents scale back their assessment of their children's maturity to be able to see the physician alone as time passes. In this regard, we note that data from other health care scenarios suggests that in addition to encouraging pediatric health care providers to anticipate such anxiety, many of the functions of a home social worker could alleviate some of these worries [15]. Viewing transitioning as an achievable competency for youth with chronic disease, (and seeing the pediatrician as instrumental in its attainment), may be a helpful perspective in this regard [16].

## Conclusion

We join the call for "strong collaborative relationships for the benefit of young patients in transition, integrating educational, vocational, mental health, and social services" [17]. For example, because juvenile myositis entails disease-specific health care issues, transition of care within this special population could include the implementation of a "transition passport" aiming at the acquisition of sufficient disease self-management skills [18]. This would be one mechanism by which adolescents with special health care needs would be less likely to "hang out" longer in pediatric care than their peers without special needs [19]. Further research regarding health care transitioning of youth with chronic disease may benefit from stratification of respondents based upon duration of illness and age of diagnosis (i.e., time in 'sick role') relative to feelings of vulnerability, self-sufficiency, and independent living skills [20].

## List of Abbreviations

ACGME: Accreditation Council for Graduate Medical Education; IIM: idiopathic inflammatory myopathies; JM: Juvenile myositis; MRI: Magnetic resonance imaging; NIEHS: National Institute of Environmental Health Sciences; NIH: National Institutes of Health

## Competing interests

The authors declare that they have no competing interests.

## Authors' contributions

JDK was responsible for implementation of the study, coordination activities and writing. GM was responsible for implementation, coordination and data analysis. SA was responsible for design and data collection. OYJ was responsible for concept development and recruitment. HB was responsible for national recruitment and coordination. AMH was responsible for international recruitment and coordination. LGR was responsible for concept, design and analysis, PHW was responsible for concept, design, and writing. All authors read and approved the manuscript.

## Authors' Information

The views expressed in this article are those of the authors and do not reflect the official policy of the Department of Army, Department of Defense, or U.S. Government. Dr. Katz had full access to all the data in the study and takes responsibility for the integrity of the data and the accuracy of the data analysis.

## Funding Considerations

This work was supported in part by the NIEHS, NIH intramural research program. This work was supported in part by Cure JM Foundation, a nonprofit organization dedicated to the cure of juvenile myositis diseases.

## References

[B1] Berkowitz S Transitioning adolescents to adult care: putting theory into practice Minn Med 2009 92 3 42 44 19400387

[B2] Hait EJ Barendse RM Arnold JH Transition of adolescents with inflammatory bowel disease from pediatric to adult care: a survey of adult gastroenterologists J Pediatr Gastroenterol Nutr 2009 48 1 61 65 10.1097/MPG.0b013e31816d71d8 19172125

[B3] Betz CL Adolescents in transition of adult care: why the concern? Nurs Clin North Am 2004 39 4 681 713 10.1016/j.cnur.2004.07.008 15561154

[B4] Simon TD Lamb S Murphy NA Hom B Walker ML Clark EB Who Will Care for Me Next? Transitioning to Adulthood With Hydrocephalus Pediatrics 2009 124 1431 1437 10.1542/peds.2008-3834 19841113 PMC2895548

[B5] Tuchman LK Slap GB Britto MT Transition to adult care: experiences and expectations of adolescents with a chronic illness Child Care Health Dev 2008 34 5 557 563 10.1111/j.1365-2214.2008.00844.x 18796047

[B6] McDonagh JE Kelly DA Transitioning care of the pediatric recipient to adult caregivers Pediatr Clin N Am 2003 50 1561 1583 10.1016/S0031-3955(03)00131-7 14710793

[B7] White PH Shear ES Transition/job readiness for adolescents with juvenile arthritis and other chronic illness J Rheumatol 1992 33 Suppl 23 27 1534377

[B8] White PH Access to health care: health insurance considerations for young adults with special health care needs/disabilities Pediatrics 2002 110 6 Pt 2 1328 1335 12456953

[B9] Annunziato RA Emre S Shneider B Barton C Dugan CA Shemesh E Adherence and medical outcomes in pediatric liver transplant recipients who transition to adult services Pediatr Transplant 2007 11 6 578 614 10.1111/j.1399-3046.2007.00756.x 17663682

[B10] McLaughlin SE Diener-West M Indurkhya A Rubin H Heckmann R Boyle MP Improving transition from pediatric to adult cystic fibrosis care: lessons from a national survey of current practices Pediatrics 2008 121 5 e1160 1166 10.1542/peds.2007-2217 18450860

[B11] Bell L Adolescent dialysis patient transition to adult care: a cross-sectional survey Pediatr Nephrol 2007 22 5 720 6 10.1007/s00467-006-0404-z 17333004

[B12] Freed L Hudson EJ Transitioning children with chronic diseases to adult care: current knowledge, practices, and directions J Pediatr 2006 148 824 827 10.1016/j.jpeds.2006.02.010 16769396

[B13] Burke R Spoerri M Price A Cardosi A Flanagan P Survey of Primary Care Pediatricians on the Transition and Transfer of Adolescents to Adult Health Care Clinical Pediatrics 2008 47 4 347 354 10.1177/0009922807310938 18180341

[B14] Andrews NR Chaney JM Mullins LL Wagner JL Hommel KA Jarvis JN The Differential Effect of Child Age on the Illness Intrusiveness-Parent Distress Relationship in Juvenile Rheumatic Disease Rehabilitation Psychology 2009 54 1 45 50 19618702 10.1037/a0014443

[B15] Wiener LS Zobel M Battles H Ryder C Transition from a pediatric HIV intramural clinical research program to adolescent and adult community-based care services: assessing transition readiness Soc Work Health Care 2007 46 1 1 19 10.1300/J010v46n01_01 18032153 PMC2366035

[B16] Olsen DG Swigonski NL Transition to Adulthood: The Important Role of the Pediatrician Pediatrics 2004 113 3 e159 e162 10.1542/peds.113.3.e159 14993570

[B17] Cervia JS Transitioning HIV-infected children to adult care J Pediatr 2007 150 1 e1 Comment on: *J Pediatr *2006, **148**(6):824-827. 17188599 10.1016/j.jpeds.2006.09.025

[B18] Rubin K Transitioning the patient with Turner's syndrome from pediatric to adult care J Pediatr Endocrinol Metab 2003 16 Suppl 3 651 659 12795368

[B19] Burke R Spoerri M Price A Cardosi A Flanagan P Survey of Primary Care Pediatricians on the Transition and Transfer of Adolescents to Adult Health Care Clinical Pediatrics 2008 47 4 347 354 10.1177/0009922807310938 18180341

[B20] Sawin KJ Bellin MH Roux G Buran CF Brei TJ The experience of self-management in adolescent women with spina bifida Rehabil Nurs 2009 34 1 26 38 19160922 10.1002/j.2048-7940.2009.tb00245.x

